# Cytomegalovirus-Specific T Cell Epitope Recognition in Congenital Cytomegalovirus Mother-Infant Pairs

**DOI:** 10.3389/fimmu.2020.568217

**Published:** 2020-11-24

**Authors:** Emma C. Materne, Daniele Lilleri, Francesca Garofoli, Giuseppina Lombardi, Milena Furione, Maurizio Zavattoni, Laura Gibson

**Affiliations:** ^1^University of Massachusetts Medical School, Worcester, MA, United States; ^2^Unità Operativa Complessa (UOC) Laboratorio Genetica - Trapiantologia e Malattie Cardiovascolari, Fondazione Istituto di Ricovero e Cura a Carattere Scientifico (IRCCS) Policlinico San Matteo, Pavia, Italy; ^3^Neonatal Unit and Neonatal Intensive Care Unit, Fondazione Istituto di Ricovero e Cura a Carattere Scientifico (IRCCS) Policlinico San Matteo, Pavia, Italy; ^4^Molecular Virology Unit, Microbiology and Virology Department, Fondazione Istituto di Ricovero e Cura a Carattere Scientifico (IRCCS) Policlinico San Matteo, Pavia, Italy; ^5^Department of Medicine, UMass Memorial Medical Center, Worcester, MA, United States; ^6^Department of Pediatrics, UMass Memorial Medical Center, Worcester, MA, United States

**Keywords:** cytomegalovirus, T cell, immune escape, epitope, congenital infection, mother-infant pairs, immunology

## Abstract

**Background:** Congenital cytomegalovirus (cCMV) infection is the most common infection acquired before birth and from which about 20% of infants develop permanent neurodevelopmental effects regardless of presence or absence of symptoms at birth. Viral escape from host immune control may be a mechanism of CMV transmission and infant disease severity. We sought to identify and compare CMV epitopes recognized by mother-infant pairs. We also hypothesized that if immune escape were occurring, then one pattern of longitudinal CD8 T cell responses restricted by shared HLA alleles would be maternal loss (by viral escape) and infant gain (by viral reversion to wildtype) of CMV epitope recognition.

**Methods:** The study population consisted of 6 women with primary CMV infection during pregnancy and their infants with cCMV infection. CMV UL83 and UL123 peptides with known or predicted restriction by maternal MHC class I alleles were identified, and a subset was selected for testing based on several criteria. Maternal or infant cells were stimulated with CMV peptides in the IFN-γ ELISpot assay.

**Results:** Overall, 14 of 25 (56%; 8 UL83 and 6 UL123) peptides recognized by mother-infant pairs were not previously reported as CD8 T cell epitopes. Of three pairs with longitudinal samples, one showed maternal loss and infant gain of responses to a CMV epitope restricted by a shared HLA allele.

**Conclusions:** CD8 T cell responses to multiple novel CMV epitopes were identified, particularly in infants. Moreover, the hypothesized pattern of CMV immune escape was observed in one mother-infant pair. These findings emphasize that knowledge of paired CMV epitope recognition allows exploration of viral immune escape that may operate within the maternal-fetal system. Our work provides rationale for future studies of this potential mechanism of CMV transmission during pregnancy or clinical outcomes of infants with cCMV infection.

## Introduction

Congenital cytomegalovirus (cCMV) infection is the most common infection acquired before birth, with an incidence of nearly 0.7% of live births in the United States (1). Of all infected infants with or without CMV symptoms at birth, almost 20% will develop permanent neurodevelopmental effects such as sensorineural hearing loss, motor disability, cognitive delay, or visual impairment (1). While current strategies to prevent transmission consist of behavioral modifications during pregnancy, there is no CMV vaccine or other intervention to lower the risk of transmission from mother to fetus or reduce severity of disease in affected infants. More effective prevention and treatment strategies are needed, informed by a deeper understanding of virus interaction with maternal and fetal hosts during early congenital infection and of mechanisms underlying CMV transmission.

Most studies of CMV-specific T cell responses have been conducted in immunocompetent adults with chronic infection or in solid organ or hematopoietic stem cell transplant recipients with reactivation or donor-derived primary infection (2–5). CMV UL83 (pp65) and UL123 (IE1) are known immunogenic proteins in these populations and have been used as antigens to study T cell responses in infants with cCMV infection (6–11). CMV-specific CD8 T cells have been detected in fetuses as early as 22–28 weeks gestation (6, 8, 12), and were characterized by oligoclonal expansion and late differentiation phenotype similar to adults (6, 8). While these responses can increase in frequency and breadth of CMV antigen recognition over time (8), their function may be suboptimal (10, 13).

While these studies have provided essential details characterizing CMV immunity in infants, none have examined the dynamic interface between CMV and host immunity within the maternal-fetal system as represented by mother-infant pairs. An example of host-pathogen dynamics is immune escape, whereby an organism thwarts the ongoing host innate or adaptive immune response or “pressure” via genomic mutations that diminish the efficacy of recognition. For viruses, such mutations likely take the form of non-synonymous changes in amino acid residues within or flanking MHC binding sites, which in turn cause modified proteolysis, disruption of the epitope, and ultimately lack of optimal T cell recognition.

CD8 T cell escape has been reported for several RNA viruses in adults, including HIV and hepatitis C and D viruses, particularly during early acute infection (14–18). Some studies have examined viral immune escape in congenital infection, primarily HIV (19–23). Of note, Sanchez-Marino et al. (20, 24) showed that maternal viral escape populations can be transmitted to the fetus, variant amino acid sequences incur viral fitness cost, and infant HIV-specific CD8 T cells can exert selective pressure on subsequent viral evolution. They also showed that the fate of transmitted escape populations depends on mother-infant concordance for MHC class I alleles restricting relevant CD8 T cell responses: mutations persist and allow continued viral escape in infants with shared alleles, while fitness-cost mutations revert to wild type in those with non-shared alleles. Previous studies have suggested that CMV populations may be as diverse as RNA viruses (25, 26), raising the possibility that CMV genomic variants can bypass protective maternal immunity, transmit to the fetus, and influence severity of disease.

We conducted a pilot study to compare CMV epitope-specific CD8 T cell responses in women with primary CMV infection during pregnancy and their infants with congenital CMV infection. The goals of the study were to identify T cell response signatures consistent with viral immune escape and to provide new information about CMV-specific T cell peptide recognition at early stages of CMV infection. We hypothesized that responses restricted by shared HLA alleles would show maternal loss (by viral escape) and infant gain (by viral reversion to wildtype) of CMV epitope recognition.

## Materials and Methods

### Subject Population

Pairs of mothers with primary CMV infection during pregnancy and their infants with cCMV infection (*n* = 6) were enrolled at Fondazione IRCCS Policlinico San Matteo in Pavia, Italy. Written informed consent was obtained from adult participants and from parents of infants. Characteristics of the subject population are shown in Table 1.

**Table 1:** Characteristics of mother-infant pairs.

**Mother**				**Infant**		
**Pair**	**Time since onset (days)**	**Trimester of onset**	**Viral load (GE/10 μL WB)**	**Age at first study (days as noted or months)**	**Time since maternal onset (days)**	**Viral load (GE/10μL WB)**
124	13	3	500	1 d	53	1000
	45		0	3.5		100
				26		0
125	45	2	10	4.5	170	3
	170		0			
127	128	1	3	2 d	317	50
	212		3	6		0
	257		3	15		0
128	69	1	0	8	210	3
				14.5		0
				26		0
129	65	3	–	1 d	94	10
				6		10
131	83	2	–	3 d	183	3
	118		–	40		3
	186		–			

Maternal primary CMV infection was determined by recent CMV-specific IgG seroconversion, presence of CMV-specific IgM and low IgG avidity, and/or presence of CMV nucleic acid detection by PCR amplification. Timing of fetal infection was not known. cCMV infection was diagnosed by urine PCR at birth. Five infants were asymptomatic at birth, and one (infant 129) had chorioretinitis that subsequently resolved. All infants were asymptomatic at the final follow-up visit (median 3 years, range 2–6 years).

Samples for the study consisted of non-antigen-specifically expanded and viably cryo-preserved maternal CD8 T cells (PBMC were not available) and infant peripheral blood mononuclear cells (PBMC). Maternal CD8 T cell lines were generated by incubating PBMCs in R10 medium with bi-specific anti-CD3, 4b (final 2.5 mg/mL) (8, 27) and stimulating with irradiated allogeneic PBMCs and anti-CD3 every 2–3 weeks for a maximum of 6 weeks, then were cryopreserved (8, 27). Cell lines were >80% CD8+ as determined by flow cytometry. Infant PBMCs were cryopreserved directly.

### CMV Peptides

CMV UL83/pp65 (*n* = 16) and UL123/IE1 (*n* = 12) 9- to 11-mer peptides predicted to bind known maternal MHC class I alleles were selected for testing based on previous studies, Immune Epitope Database and Analysis Resource (IEDB) percentile rank, BIMAS, and SYFPEITHI binding affinity scores in prediction algorithms, and number of cell available for study. Peptides at >95% purity were obtained from New England Peptide Inc. (Gardner, MA). Lack of sufficient cells precluded confirming HLA restriction of epitopes using targets expressing or matched at single HLA alleles.

### IFN-γ ELISpot Assay

The ELISpot assay was used to measure CMV peptide-specific IFN-γ release (Human IFN-γ ELIspot Ready-SET-Go! from FisherScientific, Cambridge MA). Samples from mother-infant pairs were thawed simultaneously and stimulated with the same peptides in concurrent assays.

Wells of 96 well flat-bottom plates (Millipore, Bedford MA) were washed initially with 35% ethanol in sterile water, then 3 times with PBS without allowing the membranes to dry. Diluted (1:250) capture antibody (100 μl) was added to each well and plates were stored overnight at 4°C. Wells were then washed twice with 200 μl DPBS, blocked with T cell medium, and incubated at 37°C for 15 min.

**Figure 1:**
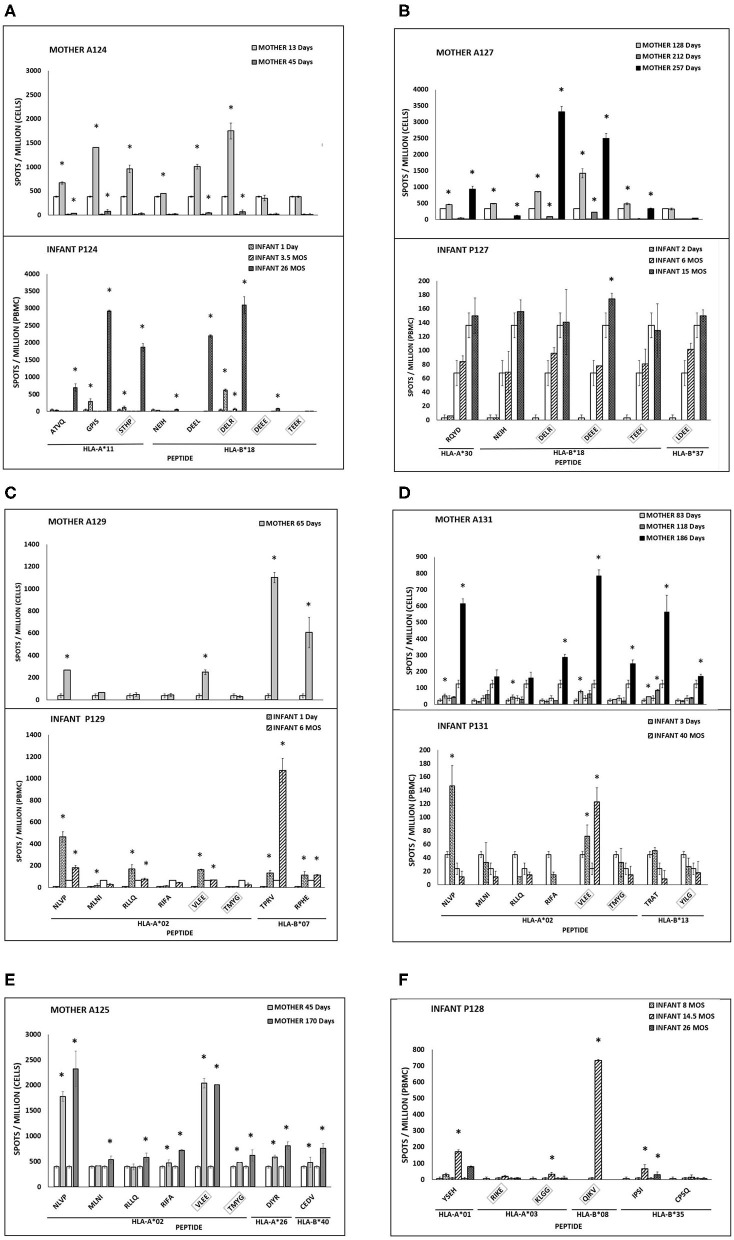
**(A–F)** CMV peptide-specific responses in mother-infant pairs. IFN-γ secreting cells are expressed as spots per million PBMC (infants) or T cells (mothers). Asterisk indicates significant response. Clear bars indicate negative control for each time point. UL83 (no box) or UL123 (box) peptides (first 4 amino acids in sequence) with HLA restricting alleles. Infant 125 and mother 128 with no detectable responses are not shown.

Cells samples were thawed in a 37°C water bath, washed with medium spun at 1,500 rpm, counted, and diluted to 1 × 10^6^ cells/ml. A total of 10^5^ cells were incubated with 0.5 μg of peptide (final 10.0 μg/mL) in each well. Mother and infant plates were prepared simultaneously. Each timepoint had at least two technical replicates, and included positive (≥2 wells of cells with PHA 50 μl at 1 mg/mL; Gibco/Life Technologies), and negative (≥4 wells of cells in medium only) (4) controls, except mother 125 at timepoint 170 days (**Table 3B**, Figure 1E) that did not have a separate negative control. Plates were incubated at 37°C for 24 h.

After 24 h, plates were washed with DPBS and then DPBS with Tween immediately prior to addition of biotinylated mAb (1:250) (FisherScientific, Cambridge MA) then incubated for 2 h. Avidin peroxidase (1:250) (FisherScientific, Cambridge MA) was added and plates were incubated for 1 h. Finally, plates were washed, peroxidase substrate (FisherScientific, Cambridge MA) was added, and the reaction was stopped after no more than 15 min. Spots were counted using a CTL Immunospot plate reader.

### HLA Typing of Infants

MHC class I alleles were known for mothers at the start of the study. Using an aliquot of cells obtained at the time of thawing for the ELISpot assay, BLCLs infants were generated from EBV-transformed PBMCs grown in enriched RPMI 1640 media (FisherScientific, Cambridge MA) (28). Infant MHC class I alleles were determined from BLCLs using a PCR-based method (University of Massachusetts Tissue Typing Laboratory, Worcester, MA).

### Data Analysis

Epitope recognition patterns for CMV-specific CD8 T cells restricted by shared and non-shared HLA alleles were compared between mothers and infants. Number of spots per million cells was calculated. Responses >2 standard deviations above the negative control were considered significant (7, 8, 29).

## Results

### Novel CMV Peptides Elicited T Cell Responses in Mothers and Infants

Predicted to be CMV epitopes based on maternal HLA alleles, a total of 28 UL83 (pp65) or UL123 (IE1) peptides were tested for CD8 T cell responses in 6 mother-infant pairs, of which 25 (89%) elicited detectable responses in the overall cohort (Table 2).

**Table 2:** CMV peptides tested for T cell responses in 6 mother-infant pairs.

**HLA restriction**	**CMV protein**	**Amino acid sequence**	**Mother**	**Infant**	**Novel (adults)**	**Novel (infants)**
A*01	UL83	YSEHPTFTSQY				X
A*02	UL83	NLVPMVATV				
		MLNIPSINV				X
		RLLQTGIHV				X
		RIFAELEGV				
	UL123	VLEETSVML				
		TMYGGISLL				
A*03	UL123	RIKEHMLKK				
		KLGGALQAK				X
A*11	UL83	ATVQGQNLK				X
		GPISGHVLK				X
	UL123	STHPMVTRSK				X
A*26	UL83	DIYRIFAEL				
A*30	UL83	RQYDPVAAL				
B*07	UL83	TPRVTGGGAM				
		RPHERNGFTVL				X
B*08	UL123	QIKVRVDMV				
B*13	UL83	TRATKMQVI				
	UL123	YILGADPLRV				
B*18	UL83	NEIHNPAVF			X	X
		DEELVTTER			X	X
	UL123	DELRRKMMY				X
		DEEEAIVAY				X
		TEEKFTGAF			X	
B*35	UL83	IPSINVHHY				X
		CPSQEPMSIYVY				
B*37	UL123	LDEERDKVL				
B*40	UL83	CEDVPSGKL				

*Shaded boxes, CMV-specific response detected in at least one mother or infant in the cohort. X, novel peptides not previously reported as CD8 T cell epitopes in adults or infants*.

Of the 25 peptides, 21 (84%) were recognized by mothers with primary CMV infection during pregnancy: 3 (14%) novel and 18 (86%) previously reported in adults. The 3 novel peptides were identified in 2 B18 mothers: B18-NEIH-UL83 and B18-DEEL-UL123 for mother 124 (and her infant), and B18-NEIH-UL83 and B18-TEEK-UL123 in mother 127 (although B18-DEEL-UL123 could not be tested due to insufficient cells).

Similarly, of the 25 peptides, 17 (68%) were recognized by infants with cCMV infection: 13 (76%; 8 UL83 and 5 UL123) novel and 4 (24%; 2 UL83 and 2 UL123) previously reported in infants (8).

For the overall cohort of mother-infant pairs, 14 of 25 (56%) recognized peptides were not previously reported as CD8 T cell epitopes: 11 in infants only, 1 in a mother only (B18-TEEK-UL123), and 2 in both groups (B18-DEEL-UL123 and B18-NEIH-UL83).

### CMV Peptide Recognition Pattern Consistent With CMV Immune Escape

Longitudinal samples were available for 3 of 6 mother-infant pairs. Of these three, pair 124 showed a CD8 T cell recognition pattern against shared-HLA B18-restricted CMV peptide NEIH-UL83 consistent with the hypothesized viral escape model of CMV transmission (Table 3A, Figure 1A).

**Table 3:** **(A)** Longitudinal CD8 T cell peptide recognition in pair 124 sharing HLA-A*11 and -B*18. **(B–F)** Longitudinal CD8 T cell peptide recognition in pairs 125, 127, 128, 129, 131.

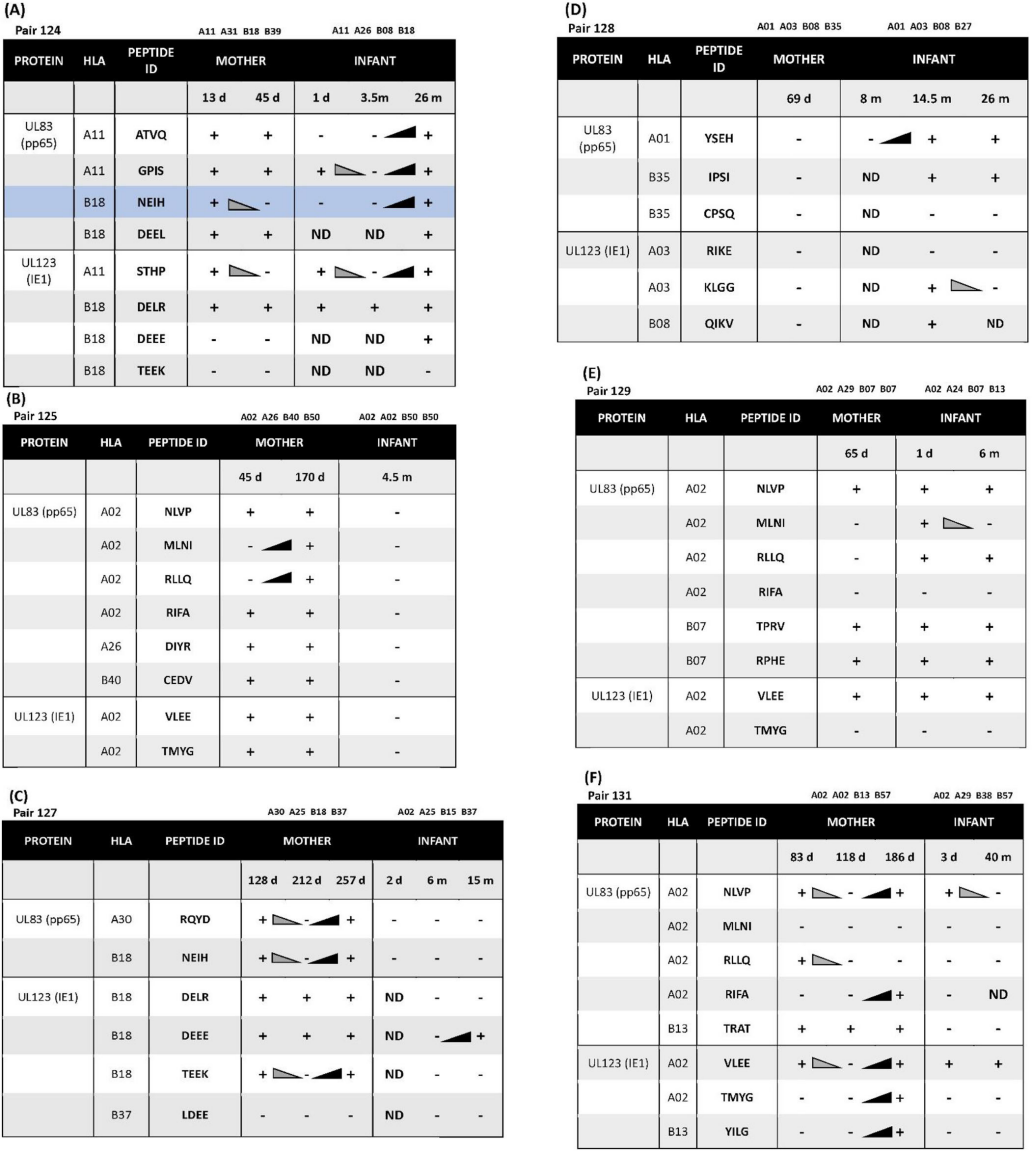

*Loss (gray wedges) and gain (black wedges) of T cell responses as indicated. +, significant response detected in IFN-γ ELISpot assay (see Figure 1 for quantitative data). –, no response detected; ND, not done due to insufficient cells available*.

**Figure 2:**
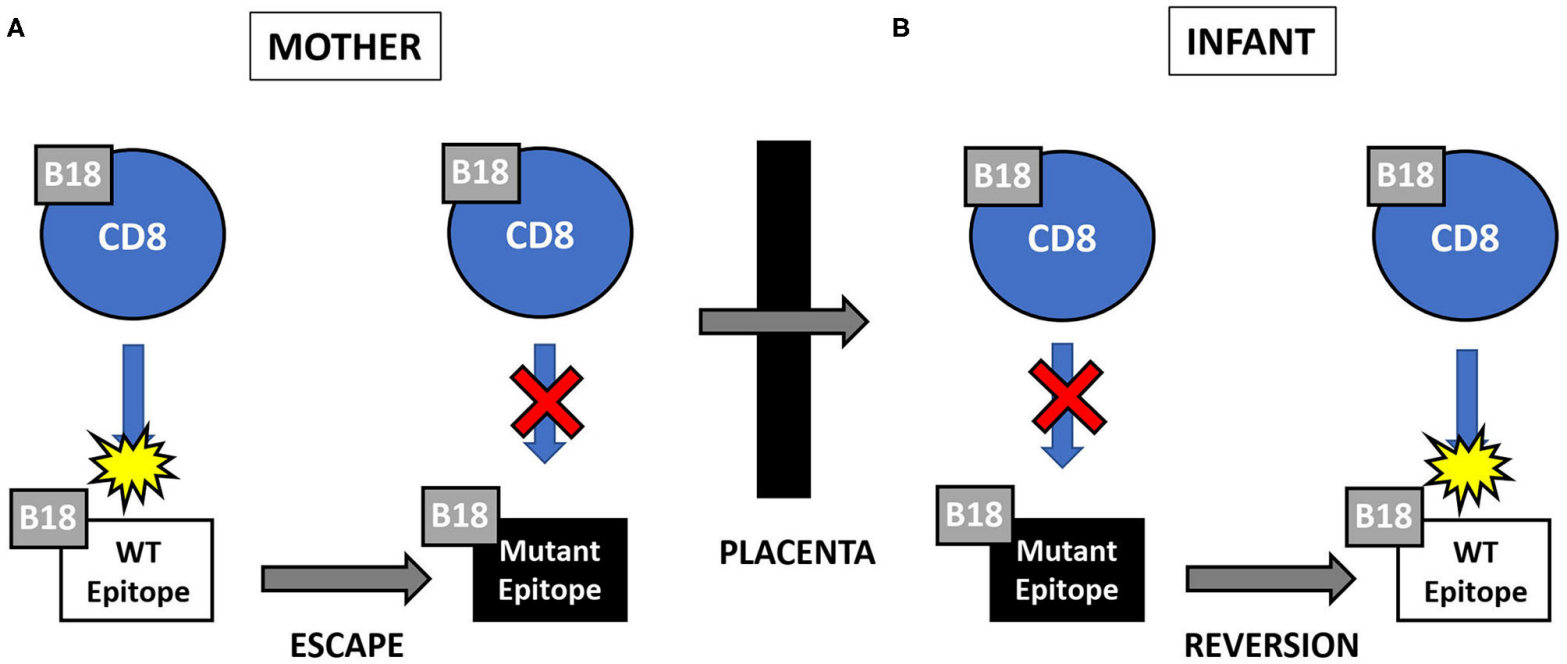
**(A,B)** Hypothesized pattern of viral escape and reversion to wild type (WT) in pair 124. CD8 T cell responses to the HLA-B18-restricted CMV epitope NEIH. **(A)** Viral mutation (or “escape”) in the presence of maternal CD8 T cell response to wild type epitope resulting in loss of anti-WT response. **(B)** Viral reversion to WT in the absence of fetal CD8 T cell response to mutant epitope resulting in gain of anti-WT response.

Mother 124 responded to this peptide at 13 but not 45 days after onset of primary CMV infection. In contrast, infant 124 did not respond to this peptide at 1 day or 3.5 months of age, but the response was detected at 26 months of age. This maternal loss and infant gain of response to a CMV epitope restricted by a shared HLA allele would be expected with viral immune escape and reversion to wildtype, respectively. A model of this viral escape pattern is outlined (Figure 2).

### Patterns of UL83- or UL123-Specific T Cell Responses Among Individual Subjects and Mother-Infant Pairs

#### Infant CMV-Specific Peptide Responses Not Predicted by HLA Type

Two infants showed peptide-specific T cell responses that were not expected based on their HLA types. Infant 127 was HLA-A02, -A25, -B15, and -B37, but only responded to a B18-restricted peptide (B18-DEEE-UL123) at 15 months of age and did not respond to the B37-restricted peptide at any timepoint (Table 3C, Figure 1B). Similarly, infant 128 was HLA-A01, -A03, -B08, and -B27, but responded to a B35-restricted peptide (B35-IPSI-UL83) at 14.5 and 28 months of age (Table 3D). Notably, the response was at a lower frequency compared to either B08- or A01-restricted peptides (Figure 1F).

#### Longitudinal Gain of Peptide Responses

Mother 131 showed increased breadth of peptide recognition over time. At 83 days after onset of CMV infection, responses to 4 peptides (A02-NLVP-UL83, A02-RLLQ-UL83, B13-TRAT-UL83, and A02-VLEE-UL123 were detected. By 186 days, the response had expanded to include 3 additional peptides (A02-RIFA-UL83, A02-TMYG-UL123, and B13-YILG-UL123) (Table 3F).

Infant 124 also showed expansion of peptide recognition. At 1 day after birth, responses to 3 peptides (A11-GPIS-UL83, A11-STHP-UL123, and B18-DELR-UL123) were detected. By 26 months, responses had expanded to 7 of the 8 peptides tested (Table 3A).

#### Longitudinal Loss of Peptide Responses

Three infants (128, 129, and 131) and two mothers (124 and 131) showed loss of detectable responses to some peptides while responses to others remained stable (Table 3, Figure 1). Infant 128 had detectable responses to B35-IPSI-UL83 at 14.5 and 26 months of age, but loss of response to A03-KLGG-UL123 over the same interval (Table 3D, Figure 1F). Infant 129 had loss of detectable responses to A02-MLNI-UL83 between 1 day and 6 months of age (Table 3E, Figure 1C). Infant 131 had the same pattern for A02-VLEE-UL123 and A02-NLVP-UL83, respectively, at 3 days and 40 months of age (Table 3F, Figure 1D). Mother 124 had sustained detectable responses to 4 of 8 peptides tested at 13 and 45 days after infection onset, but loss of responses to B18-NEIH-UL83 and A11-STHP-UL123 over the same interval (Table 3A). Mother 131 had detectable responses to B13-TRAT-UL83 at all three timepoints sampled, but loss of responses to A02-RLLQ-UL83 between 83 and 118 days after infection onset (Table 3F).

## Discussion

We report results of a pilot study comparing CMV epitope-specific CD8 T cell responses in women with primary CMV infection during pregnancy and their infants with congenital infection. The goals of the study were to identify a hypothesized pattern of T cell responses (maternal loss and infant gain) consistent with viral immune escape and to describe CMV peptide recognition in mother-infant pairs. We identified this pattern in 1 of 3 mother-infant pairs with longitudinal samples and defined novel CMV epitopes that elicited T cell responses in the overall cohort. This work presents new information about CMV-specific T cell peptide recognition at early stages of primary or congenital infection in populations for whom previous studies are limited. While CMV genome sequencing and therefore direct evidence of viral mutation affecting T cell epitope recognition was not possible, our results support further studies of immune selection pressure on viral populations.

Several new CMV peptides were identified as targets of CD8 T cells, but the number differed between mothers and their infants: Few (14%) of the maternal epitopes and most (81%) of the infant epitopes had not been reported previously in adults with chronic or infants with cCMV infection, respectively. Of note, the 3 novel maternal epitopes were B18-restricted, one of which (B18-NEIH-UL83) elicited a detectable response in both HLA-B18 women in the cohort. In line with previous studies (6, 8) responses to CMV peptides were detected very early after birth.

As a primary mediator of immune protection, the interface between T cells and viral epitopes within the maternal-fetal system is an essential but inadequately understood aspect of CMV pathogenesis. Few studies have examined CMV-specific CD8 T cells in adults with primary or infants with congenital CMV infection, although some (10, 13, 30, 31) have noted differences in responses between primary and chronic infection and between infants and adults. CD8 but not CD4 T cell responses are similar in young children and adults during congenital or primary CMV infection (31) suggesting that stimulation of CD8 T cells is a key feature of immune protection at this early phase. Moreover, the role of CD8 T cells in controlling and monitoring as a biomarker for CMV reactivation has been described in diverse patient populations (32–35). As a result, CMV peptides that induce CD8 T cell responses have been used in multiple CMV vaccine and adoptive T cell platforms (36, 37), for which young children may be an optimal target (38). A broad repertoire of protective CMV epitopes that elicit T cell responses depending on phase of infection, age, or immune function will facilitate the design of a CMV vaccine or other immune-based interventions.

Our study also revealed differential patterns of CD8 T cell responses to CMV peptides over time in mother-infant pairs. While this range of patterns may be in part due to technical issues such as limitations in epitope prediction models, maternal samples with differential expansion of maternal CMV-specific T cells (despite non-specific stimulation *in vitro*) or insufficient antigen-presenting cells, or low cell viability (unlikely when responses to other peptides were detected at the same timepoints), our experimental approach sought to minimize these effects by preparing cells and performing ELISpot assays for each mother and her infant simultaneously. Alternatively, responses detected for the first time at a later timepoint may have been present earlier but were undetectable by the ELISpot assay.

An increase in the number of CMV peptides recognized over time was observed in pairs 124 and 131 for infant and mother, respectively. Gibson et al. similarly demonstrated broadening of peptide targeting in infants with cCMV infection, primarily to UL123 (IE1) (8). Possible explanations for widening antigenic breadth of CD8 T cells over time include exposure to CMV proteins during reactivation or persistent replication in tissue compartments, CMV re-infection, CMV genome mutation and altered amino acid sequences, or expansion of CMV-specific CD4 T cells and helper function (9, 31, 39–42).

In contrast, loss of CD8 T cell responses to a single CMV peptide was observed for each of infants 128, 129, and 131, and mothers 124 and 131. A potential factor is CD8 T cell exhaustion associated with high antigen exposure, characterized by a progressive loss of effector functions, increased expression of inhibitory receptors such as PD-1, and inability to transition into memory, and described in many chronic infections including HIV, HCV, EBV, and CMV (13, 43, 44). Moreover, T cell exhaustion has been shown to develop within 30 days after infection in several animal and human viral infections (45–47). While examining PD-1 and other markers of exhaustion was not feasible, the infants in our cohort were likely CMV-infected long enough for exhaustion to develop. Loss of single CMV peptide recognition could also be attributed to viral escape from infant CD8 T cell selective pressure as described for congenital HIV infection (20, 24). In these studies, HIV epitopes with single amino acid substitutions were identified in infants during the first year of life but not in maternal plasma and reverted to wild type coincident with loss of CD8 T cell response, suggesting that immune pressure in the infant led to their generation. Our study is the first to report loss of CMV peptide recognition consistent with this process in infants with cCMV infection.

Two infants in the cohort (127 and 128) had detectable CD8 T cell responses to CMV peptides (DEEE-UL123 and IPSI-UL83, respectively) not predicted to bind their MHC class I alleles, which was not observed in mothers since peptides were chosen in part based on predicted binding. Of note, neither peptide had been previously reported as a CD8 T cell epitope in infants. DEEE-UL123 is restricted by HLA-A01 (3) and has a high binding affinity score for HLA-B18, but infant 127 does not express either of these alleles. B35-IPSI-UL83 is included in the QuantiFERON®-CMV assay (Qiagen) used to monitor CMV-specific CD8 T cells in transplant recipients (2), but infant 128 does not express the restricting HLA allele B35.

This discrepancy between HLA restriction and detected T cell response could be explained by MHC promiscuity and/or limitations of epitope prediction models. While uncommon (compared to CD4 T cells) due to the restrictive size of the MHC class I binding site (48), promiscuous binding of CD8 T cell epitopes by MHC class I molecules has been described (49). At least half of HIV or EBV epitopes elicit a T cell response in subjects lacking the reported restricting HLA allele (49, 50). Similarly, EBV and CMV epitopes can be presented by the same MHC class I molecule (51). MHC class I molecules can be grouped into MHC supertypes based on similarities in binding pocket characteristics (52). For example, MHC class I subtypes B18 and B37 belong to the B44 supertype in which the B pocket binds acidic residues and the F pocket binds aromatic, aliphatic, and hydrophobic residues (52), and therefore could present the same peptide. The potential for presentation by multiple HLA alleles benefits CMV interventions based on T cell stimulation with subsets of CMV peptides and use across diverse target populations (36, 37).

A pattern of epitope recognition that may support our hypothesis of viral escape and reversion was observed in mother-infant pair 124 with concordance at the HLA restricting allele. Maternal peptide B18 NEIH-specific CD8 T cells were detected at 13 days but not at 45 days after onset of primary infection, which would be predicted for viral immune escape. In contrast, peptide B18 NIEH-specific CD8 T cells were not identified in her infant until 26 months of life, as would be expected for viral reversion to wild type over time. While the possible explanations discussed above for loss or gain of detectable responses may apply to this individual mother or infant, the overall pattern for the pair is consistent with viral escape and reversion.

As noted, several previous studies examining viral immune escape in congenital (primarily HIV) infection (19–23), have shown that CMV populations can be as diverse as RNA viruses (26, 39, 53, 54), and identified signatures of positive selection within individuals (55). These findings suggest that CMV can escape the selective pressure of maternal immunity, and that genomic variants can be transmitted to the fetus. If maternal viral escape populations are transmitted, otherwise protective early fetal responses for which the restricting MHC class I allele is shared may be inadequate as virus continues to escape the new fetal host immune system. In contrast, if the relevant restricting allele is not shared, then uncompensated fitness-cost escape mutations revert to wild type against which early fetal responses are protective. This potential mechanism renders the HLA-concordant fetus/infant at higher risk of severe or progressive disease when virus escapes both maternal and fetal immune control via mutations in critical protective epitopes, thus could at least partially explaining the variability of disease severity observed in infants with cCMV infection.

In summary, we have characterized several novel CD8 T cell CMV epitopes and illustrated a pattern of CMV immune escape in one mother-infant pair that may operate within the maternal-fetal system. Moreover, our study emphasizes that CMV escape could impact not only transmission during pregnancy and/or infant clinical outcomes, but also efficacy of proposed treatment or prevention strategies. More detailed examination of mother-infant pairs is needed to investigate host immune selection and viral escape as a relevant mechanism in congenital CMV transmission and severity, for which the reported novel CD8 T cell epitopes recognized by mother-infant pairs may be useful. While appropriate samples were not available for this study, CMV genome sequencing will be necessary to identify polymorphisms that lead to epitope amino acid substitutions and altered T cell binding. Our work provides rationale for the planning and implementation of these studies.

## Data Availability Statement

The raw data supporting the conclusions of this article will be made available by the authors, without undue reservation.

## Ethics Statement

The studies involving human participants were reviewed and approved by Fondazione IRCCS Policlinico San Matteo, Pavia, Italy. Written informed consent to participate in this study was provided by the participants' legal guardian/next of kin.

## Author Contributions

EM and LG conceived the original idea for this work, including study aims, hypothesis, and experimental design, conducted data analysis, designed figures, and wrote the manuscript. EM designed CMV-specific peptides, performed all ELIspot assays, and generated BLCL infant cell lines for HLA typing. All authors discussed the results and provided feedback on the manuscript prior to submission. All authors contributed to the article and approved the submitted version.

## Conflict of Interest

The authors declare that the research was conducted in the absence of any commercial or financial relationships that could be construed as a potential conflict of interest.

## Abbreviations

HLA: human leukocyte antigen
MHC: major histocompatibility complex.
